# Cutaneous Anthrax

**DOI:** 10.5811/cpcem.52270

**Published:** 2026-08-06

**Authors:** Steve C. Christos, Abdelhamid Mazouni

**Affiliations:** Resurrection Medical Center, Department of Emergency Medicine, Chicago, Illinois

**Keywords:** cutaneous anthrax, images, emergency medicine

## Abstract

**Case Presentation:**

A 60-year-old male from the country of Jordan presented to the emergency department with swelling, pain, black lesions on his fingertips and thumb, and a red streak up his arm. The patient had been trimming sheep wool and goat skin two weeks prior to onset of symptoms and cut his left thumb with trimming shears.

**Discussion:**

Cutaneous anthrax is caused by *Bacillus anthracis*, a Gram-positive, spore-forming rod found naturally in the soil. Populations at greatest risk include those who consume undercooked meat with contaminated spores or who live and work in rural/agricultural areas. Occupations considered to be at a higher risk include farmers, wool sorters, and veterinarians. Although cutaneous anthrax is rare in the United States (U.S.), there have been cases reported since the bioterrorism attacks in 2001 when mail laced with anthrax was sent via the U.S. Postal Service. Human infection occurs in countries where the disease is endemic in livestock. Because there are no rapidly available diagnostic tests the diagnosis is primarily clinical. It is important to consider the possibility of cutaneous anthrax in the appropriate setting, despite the rarity of cases in the U.S. High clinical suspicion should be maintained in anyone presenting with the appropriate skin findings, especially in those traveling from endemic countries (South and Central Asia, Sub-Saharan Africa, Southern and Eastern Europe, Central America, South America, and the Caribbean).

## CASE PRESENTATION

A 60-year-old male from Jordan presented to the emergency department with swelling, pain, black lesions on his fingertips and thumb, and a red streak up his arm. The patient had been trimming sheep wool and goat skin two weeks prior to onset of symptoms and cut his left thumb with trimming shears. He denied lymphadenopathy, fevers, chills, nausea, vomiting, diarrhea, cough, chest pain, shortness of breath, headache, or neck stiffness. His brother, who was with him, reported similar hand lesions.

**Image 1:**
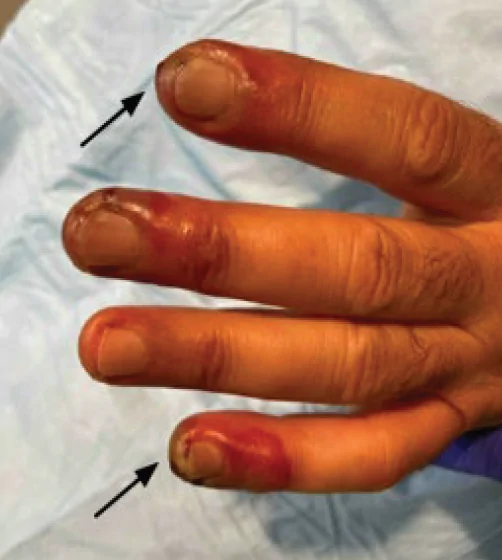
Dorsal view of the left-hand demonstrating edema and erythema of the distal aspects of the digits, with ulcerations (arrows) at the fingertips.

**Image 2:**
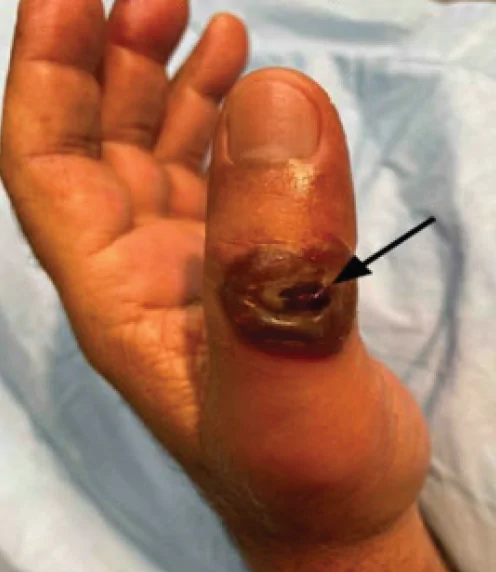
Dorsal view of the left thumb with a painless black ulcerating lesion (arrow) surrounded by edema and erythema over the interphalangeal joint.

**Image 3:**
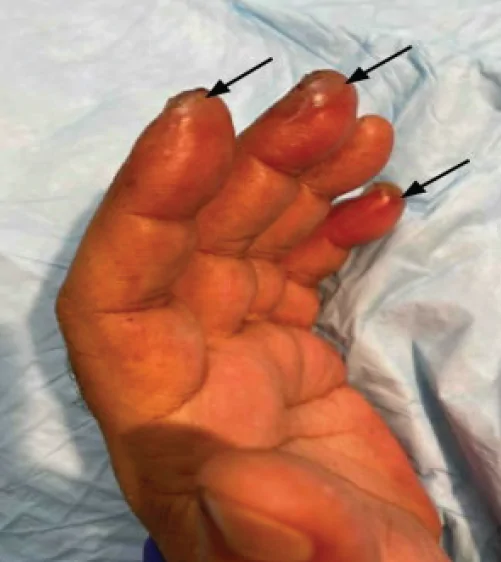
Radial view demonstrating left-hand fingertip erythema and edema (arrows).

The patient had full active and passive range of motion in all digits. There was tenderness and swelling of the distal pulp of the left fingers, along with lesions on the left hand and thumb that had a dark center. Lymphangitic streaking was present. Capillary refill was two seconds. There was no tenderness along the flexor sheath and no pain with passive extension (Images 1–3).

## DISCUSSION

Anthrax is caused by *Bacillus anthracis*, a Gram-positive rod bacteria found in soil that infects grazing animals (sheep, goats, and cattle). Humans become infected by handling animals or animal products that contain spores or via bioterrorism events. Populations at greatest risk include those who consume undercooked meat with contaminated spores and those living in rural/agricultural areas.^1^ The presentation depends on route of exposure, including inhalation of spores (inhalation anthrax), eating contaminated food or drinking water (gastrointestinal or oropharyngeal anthrax), through open skin wounds (cutaneous anthrax), injection drug use (injection anthrax), or primary anthrax meningitis. Our patient presented with classic cutaneous anthrax, the most common form of anthrax.^2^

In cutaneous anthrax, spores enter through open lesions into the subcutaneous tissue where they germinate and form toxins locally, most notably the edema toxin. Symptoms present 1–10 days after exposure with a pruritic papule, which becomes a non-pruritic vesicle that progresses to a painless ulcer with the classic black necrotic center. Cutaneous anthrax is usually limited to areas where the spores enter the skin. However, bacteria can spread hematogenously to the brain, lungs, kidneys, and spleen.

Diagnosis includes physical exam, travel history, and laboratory studies. Painless necrotic ulcers should raise suspicion for cutaneous anthrax. Routine labs include complete blood count, electrolytes, kidney function tests, liver enzymes, coagulation tests, and blood cultures. Additional studies should be sent to laboratories that evaluate anthrax such as the Laboratory Response Network (LRN). Two swabs should be collected from vesicular fluid, eschars, or ulcers and sent to an LRN lab for evaluation of Gram-stain and culture and polymerase chain reaction testing. If punch biopsy (full thickness) is performed, samples should be sent to the LRN for histopathology, special stains, and immunohistochemistry. Serology testing includes acute and convalescent serum samples and anthrax lethal factor.

*CPC-EM Capsule*

What do we already know about this clinical entity?

*Clinical presentations of anthrax vary according to mode of transmission—inhalation, gastrointestinal or oropharyngeal, cutaneous, injection, or primary anthrax meningitis.*

What is the major impact of the image(s)?

*The images reinforce key features that should raise clinical suspicion for cutaneous anthrax, including a painless ulcer with a black necrotic center.*

How might this improve emergency medicine practice?

*Early diagnosis and treatment is important as the mortality rate for untreated cutaneous anthrax is up to 20%.*

Cutaneous anthrax treatment involves oral ciprofloxacin or doxycycline. If there is extensive edema, head and neck involvement or systemic illness the patient needs to be treated with a combination of two bactericidal agents (meropenem and ciprofloxacin or levofloxacin), and one protein-synthesis inhibitor (doxycycline or minocycline). 3 Early diagnosis and treatment is important as untreated cutaneous anthrax can progress from a localized infection to widespread systemic illness including sepsis or meningitis. Mortality rate for untreated cutaneous anthrax is up to 20%.^1^
